# Novel *MAGT1* Mutation Found in the First Chinese XMEN in Hong Kong

**DOI:** 10.1155/2022/2390167

**Published:** 2022-02-14

**Authors:** Elaine Yuen Ling Au, Edmund Kwok Kwan Tung, Ricky Wai Ki Ip, Philip Hei Li

**Affiliations:** ^1^Department of Pathology, Queen Mary Hospital, Pok Fu Lam, Hong Kong; ^2^Department of Medicine, Queen Mary Hospital, Li Ka Shing Faculty of Medicine, The University of Hong Kong, Pok Fu Lam, Hong Kong

## Abstract

The availability of next-generation sequencing (NGS) helps to resolve many of the diagnostic odysseys. Common variable immunodeficiency disease (CVID) is an entity encompassing a heterogenous group of conditions with hypogammaglobulinemia, and it is a diagnosis of exclusion. In recent years, with the advances of molecular diagnostics, more and more patients have been reclassified with more defined entities after their genetic causes were found. Here, we reported a young man, who was managed as CVID since childhood, presenting with recurrent infection, hypogammaglobulinemia, and immune thrombocytopenia (ITP). Finally, more than a decade after initial presentation, gene panel testing revealed a novel mutation in the MAGT1 gene. Collectively, the genetic findings and clinical presentations confirm the diagnosis of X-linked immunodeficiency with magnesium defect and Epstein–Barr virus infection and neoplasia (XMEN). MAGT1 is an evolutionarily conserved, magnesium-specific transporter expressed in all mammalian cells that plays an essential role in magnesium homeostasis. MAGT1 also acts as an accessory protein for STT3B, as catalytic subunits of the oligosaccharyltransferase protein complex, which carries out glycan chain transfer to proteins in the endoplasmic reticulum during N-glycosylation. Glycans play an essential role in the stability, maturation, and localization in glycoproteins that are important in our immune cells' function. Mutation of the gene resulted in a rare X-linked recessive condition XMEN. The disease has complete penetrance but variable expressivity. It is mainly associated with immunodeficiency, immunodysregulation, and predisposition to EBV-associated lymphoproliferation. Extraimmune manifestations have also been reported in some patient cohorts, including hepatic and neurological abnormalities. Overall, the presentation varies among patients and overlaps with other clinical entities, in which diagnosis is challenging. Before the era of NGS, traditional workup hinges heavily on phenotype studies, followed by single-gene sequencing. The diagnostic yield is low, and a significant delay in diagnosis is common. This case illustrated the importance of early consideration of molecular studies in complex immunological cases without obvious secondary causes as an integral part of patient management.

## 1. Introduction

The magnesium transporter 1 (*MAGT1*) gene is located in chromosome Xq21.1 and has 10 exons with the predominant form encoding a 335-amino acid protein. MAGT1 is evolutionarily conserved and ubiquitously expressed. Mutations of the *MAGT1* gene result in a rare X-linked recessive condition called X-linked immunodeficiency with magnesium defect and Epstein–Barr virus (EBV) infection and neoplasia (XMEN) [1]. It has been reported in the literature [2, 3], but the clinical presentation of XMEN is variable and may overlap with other clinical entities such as common variable immunodeficiency (CVID), and therefore, diagnosis is still challenging. Here, we report the first case of Chinese XMEN in Hong Kong, with a novel mutation in the MAGT1 gene, who was previously diagnosed as CVID since childhood.

## 2. Case Vignette

A 25-year-old Chinese male student, with a prior diagnosis of CVID since childhood, was referred to the adult Immunology Clinic for continuation of care. He initially presented at 5 years old with recurrent chest infections and an episode of herpes zoster. Initial workup revealed an immunoglobulin (Ig) G level of around 500–600 mg/dl, low IgA, and absent antibody response following a pneumococcal vaccination challenge. He was diagnosed and managed as CVID with regular IVIg replacement.

Three years following his initial presentation, he was also diagnosed with immune thrombocytopenic purpura (ITP). He was treated with courses of high-dose systemic steroid and immunomodulatory doses of IVIg and remained well thereafter. However, he stopped regular IVIg replacement at age 11 as requested by his parents.

At age 24, he was admitted for an episode of ITP relapse with the lowest platelet count down to 1 × 10^9/L and was refractory to IVIg and pulse steroid therapy. He finally responded to a course of anti-CD20 therapy (rituximab) and eltrombopag. Prior to the administration of anti-CD20 therapy, his lymphocyte subset showed high B-cell counts (728/uL), with borderline low CD4 (around 400/uL). Further B-cells' immunophenotyping revealed reduced switched memory B cells and class-switched plasmablasts. Despite a borderline low CD4 count, lymphocyte proliferation by mitogen stimulation was grossly unremarkable (Supplementary Table S1).

During subsequent follow-up, there was a progressive drop in IgG levels to around 500 mg/dl, with recurrent chest infections despite on azithromycin prophylaxis. The patient was finally resumed on regular IVIg replacement at age 25.

To elucidate the potential genetic causes, a targeted next-generation sequencing (NGS) analysis was performed with Illumina TruSight One Sequencing Panel. Analyses revealed the patient was hemizygous for a novel pathogenic mutation in the *MAGT1* gene (c.916del) and was confirmed by Sanger sequencing (Supplementary Figure S1). The mutation leads to frame shift starting at codon 306, changing leucine to cysteine, and ends in a premature termination stop codon at 8 position downstream that is expected to disrupt the gene function. The variant is rare and is not found in population databases such as gnomAD at the time of reporting.

**Figure 1:**
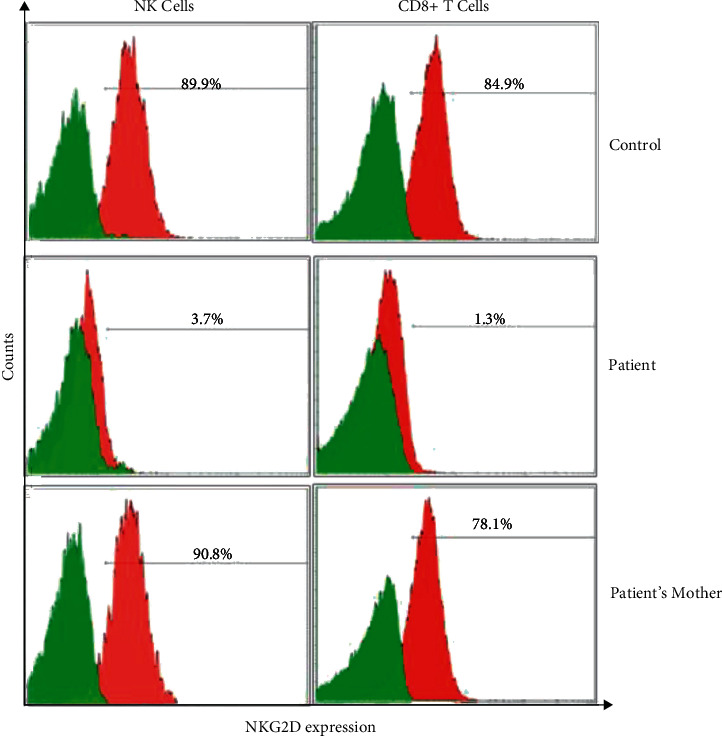
NKG2D expression in NK cells and CD8+ cytotoxic T cells.

In view of the molecular results, NKG2D expression in the patient's NK cells and CD8+ cytotoxic T cells was studied that was absent (Figure 1). EBV VCA antibodies and DNA measurement were unremarkable. His family history was unrevealing, but his mother was a confirmed carrier after genetic testing.

Collectively, the variant was classified as pathogenic, and the findings confirmed the diagnosis of XMEN.

## 3. Discussion

We report a novel *MAGT1* mutation found in the first Chinese XMEN patient in Hong Kong. *MAGT1* is an evolutionarily conserved Mg^2+^-specific transporter expressed in all mammalian cells that is essential in magnesium homeostasis. Chronic reductions in basal levels of free Mg^2+^ may contribute to the loss of NKG2D expression which is essential for antiviral and antitumor responses. In addition, *MAGT1* also acts as an accessory protein for STT3B, as catalytic subunits of the oligosaccharyltransferase protein complex, which carries out glycan chain transfer to proteins in the endoplasmic reticulum during N-glycosylation. Immune cells, in contrast to other tissues, rely exclusively on MAGT1 to facilitate asparagine (N)-linked glycosylation of specific STT3B-dependent glycoprotein that probably explains the predominant immunological aberrant manifestations of the disease [2, 4, 5].

Our patient demonstrated a decreased expression of NKG2D in his NK cells and cytotoxic T cells. He also had low serum immunoglobulins, along with impaired response to polysaccharide antigens, as reported in the literature [4]. Though he showed elevated B-cell counts, the class-switched memory B cells and plasmablasts were low. The underlying pathogenesis affecting the B-cell compartment in XMEN has not been fully elucidated yet, though the previous mouse model study showed abolished MAGT1 function caused imbalanced cation homeostasis and an impact on B-cell development [6].

In the absence of a positive family history, physicians may not be aware of this entity, and diagnosis may be delayed until complications arise. In contrast with the conventional single gene-by-gene workup which heavily relies on the characteristic phenotype, NGS serves as a powerful tool to assay gene panels or even exome/genome within a single assay that hastens the workup with enhanced yield. Knowing the underlying genetic cause has significant implications on prognostication and management, such as close surveillance of EBV-related complications and malignancies.

Workup for other family members is also important in hereditary conditions. Interestingly, the mother had normal NKG2D expression despite being a carrier. In fact, our lyonization study confirmed a pattern of X chromosome inactivation skewed towards the normal allele (Supplementary Figure S2), as similarly reported in previous studies [7–9]. Therefore, although NKG2D expression serves as a classical biomarker for XMEN, genetic studies are required for carrier detection.

## 4. Conclusion

In summary, we report a case of XMEN, with a novel mutation in the MAGT1 gene. The condition is rare, with variable clinical phenotype. While reports of further cases may help us to better delineate the phenotype spectrum, diagnosis remains challenging. Early consideration of the NGS workup may help to resolve these diagnostic odysseys.

## Supplementary Materials

Supplementary Materials Supplementary Table S1: summary of laboratory results of our patient. Supplementary Figure S1: family pedigree and DNA sequencing chromatograms of the patient and his parents. Supplementary Figure S2: X-inactivation analysis of the patient's mother (MAGT1 carrier).
