# Supplementary material for: Novel MAGT1 Mutation Found in the First Chinese XMEN in Hong Kong
Source: Case Reports Immunol. 2022 Feb 14;2022:2390167. doi: 10.1155/2022/2390167 (PMC8860550; doi:10.1155/2022/2390167)
Supplement: Supplementary Materials — Supplementary Table S1: summary of laboratory results of our patient. Supplementary Figure S1: family pedigree and DNA sequencing chromatograms of the patient and his parents. Supplementary Figure S2: X-inactivation analysis of the patient's mother (MAGT1 carrier). [file 2390167.f1.zip › 2390167.f1/Supplementary Figure S1 (1)revision.docx]

**Supplementary Figure S1**

**Family pedigree and DNA sequencing chromatograms of the patient and his parents**

**(a)**

Father (Wild type)

Mother (heterozugous)

Asymptomatic carrier

index patient

Presented with recurrent infection, hypogammaglobulineamia and ITP

**(b)**

**
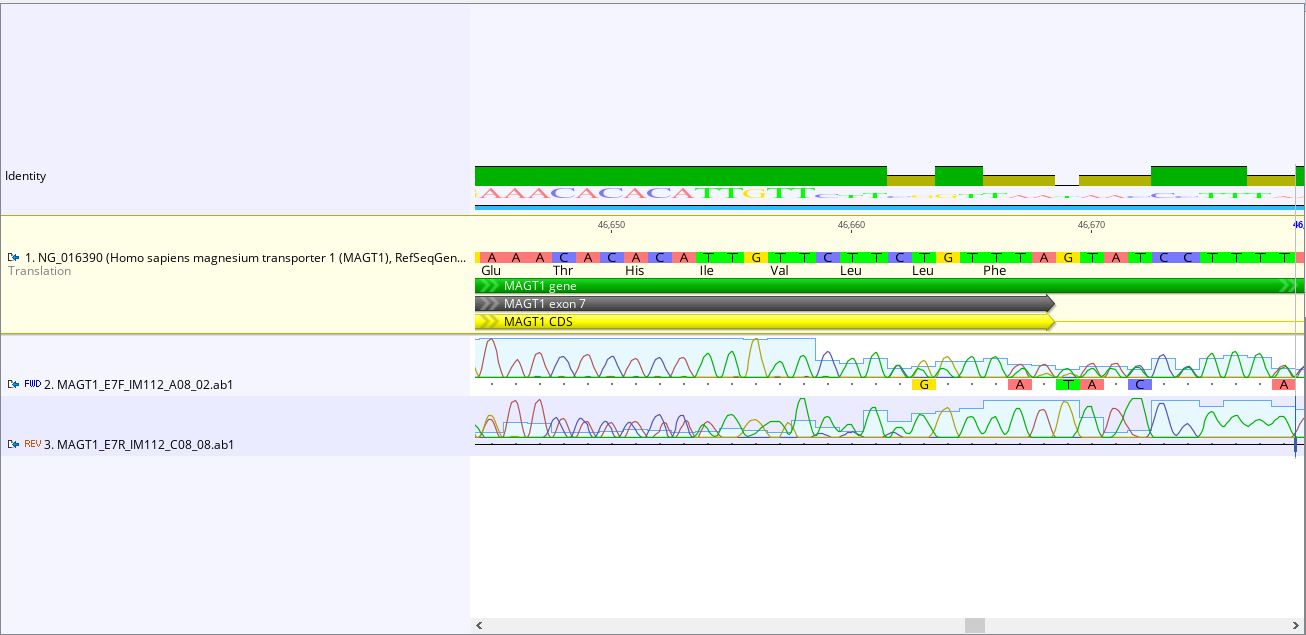

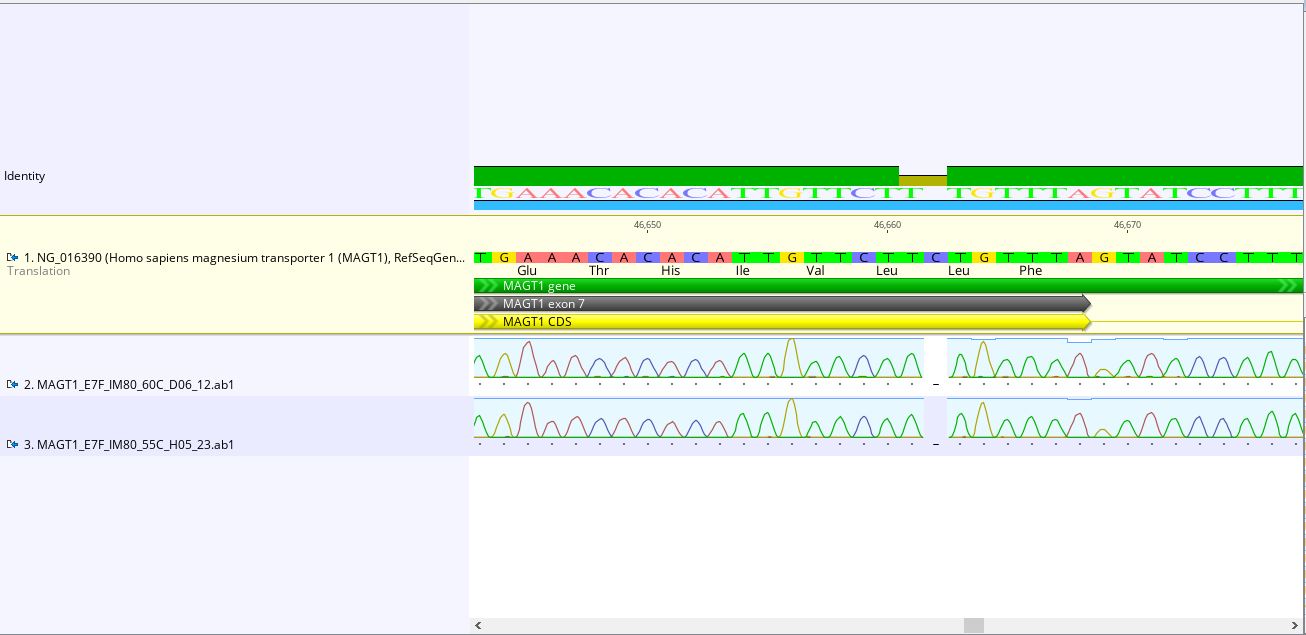

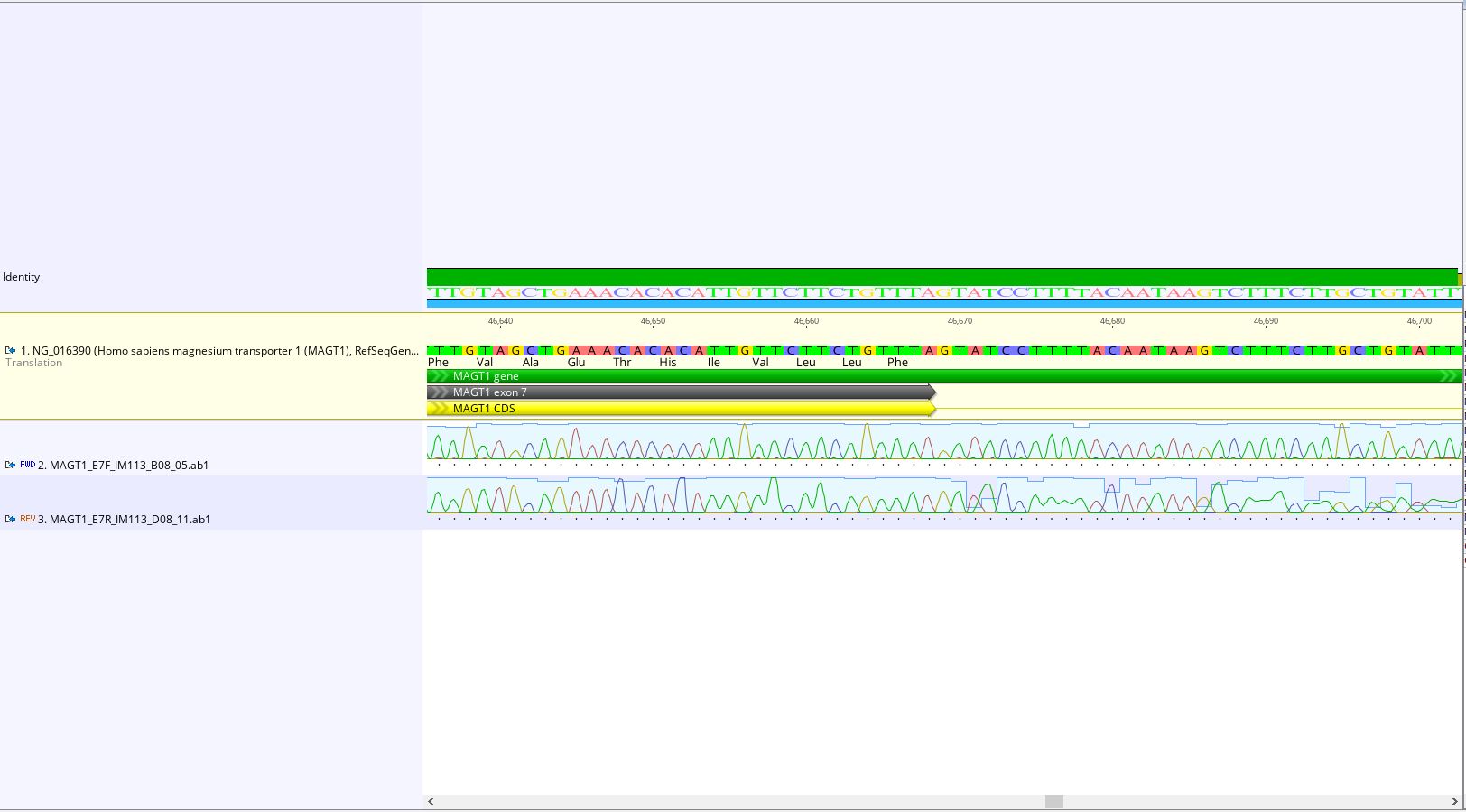
**

**Patient**

**Mother**

c.916delC

hemizygous

c.916delC

Heterozygous

**Father**

Wild Type
