# Supplementary material for: Novel MAGT1 Mutation Found in the First Chinese XMEN in Hong Kong
Source: Case Reports Immunol. 2022 Feb 14;2022:2390167. doi: 10.1155/2022/2390167 (PMC8860550; doi:10.1155/2022/2390167)

**Supplementary Figure S2**

**X-inactivation analysis of patient’s mother (Magt1 Carrier).**

HpaII -

HpaII +

XCI skewed ratio

0.0

Patient’s Mother

Magt1 Carrier

Male Control

Female Control


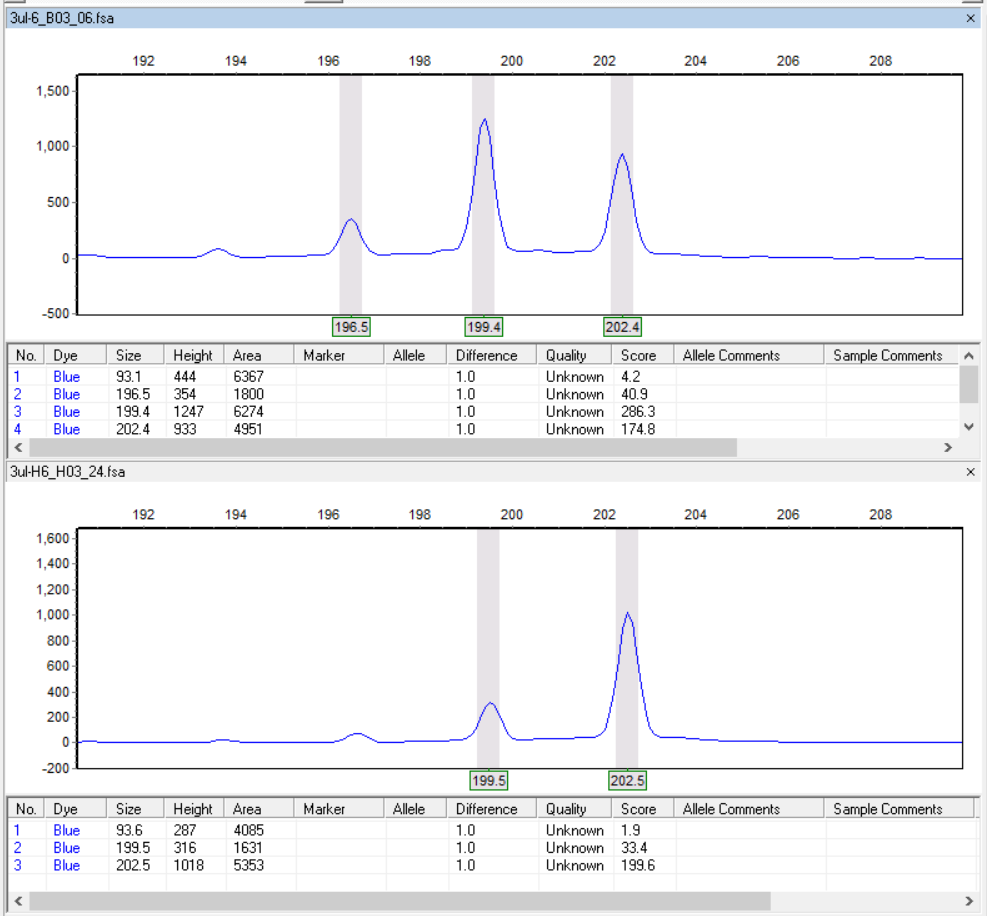

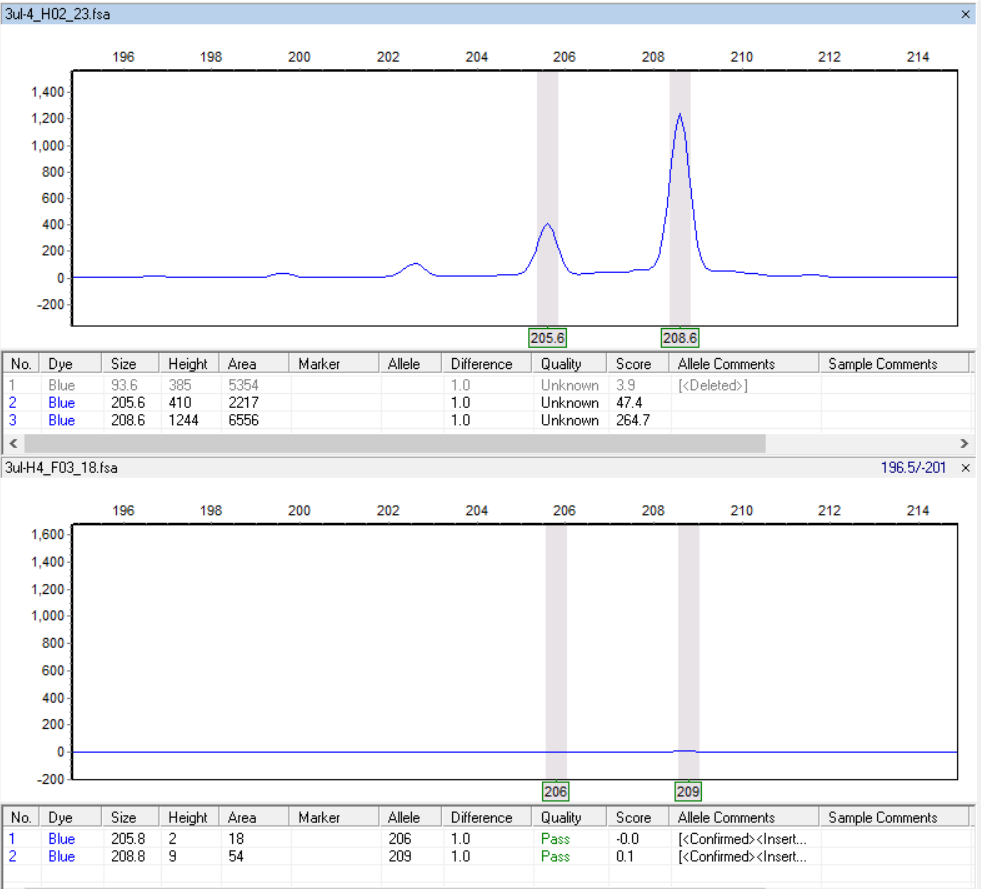


0.51


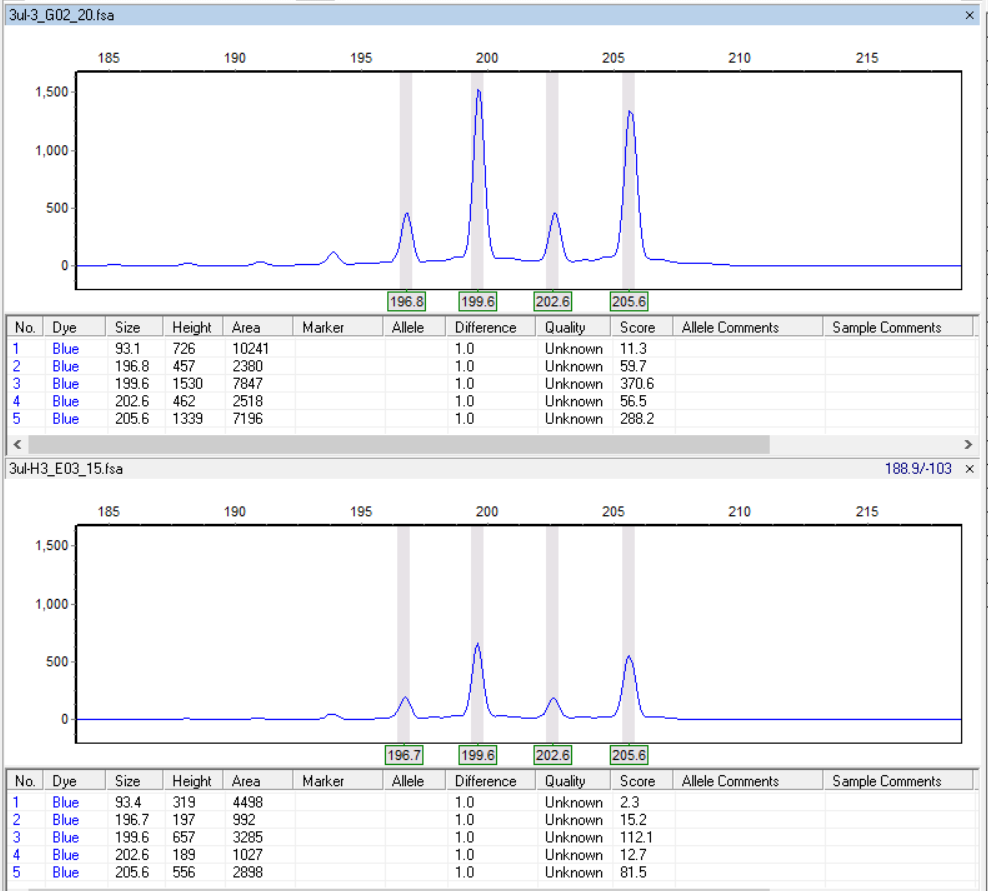

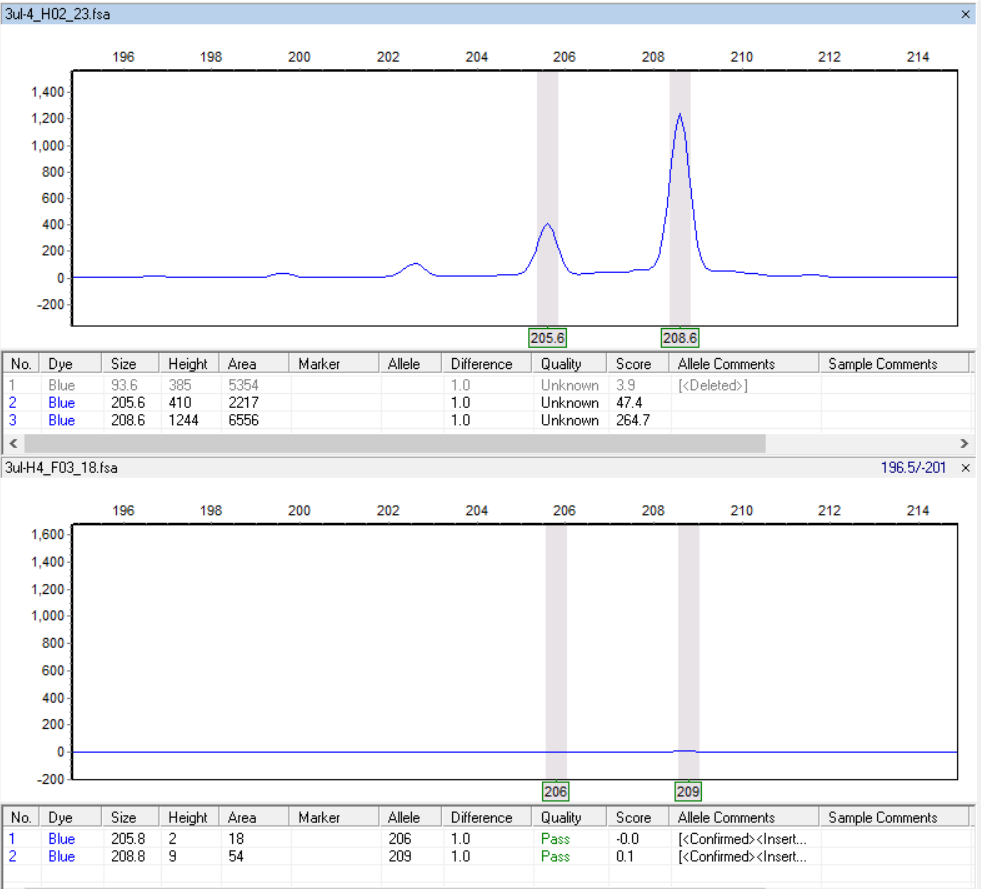

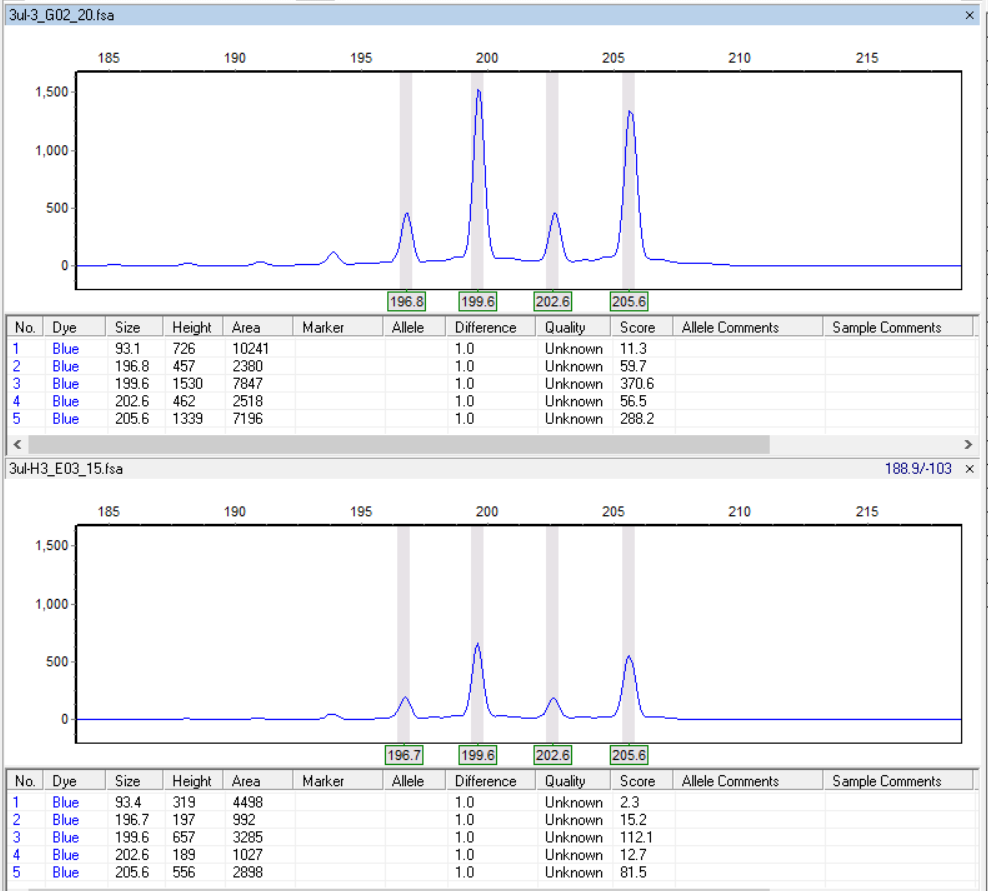

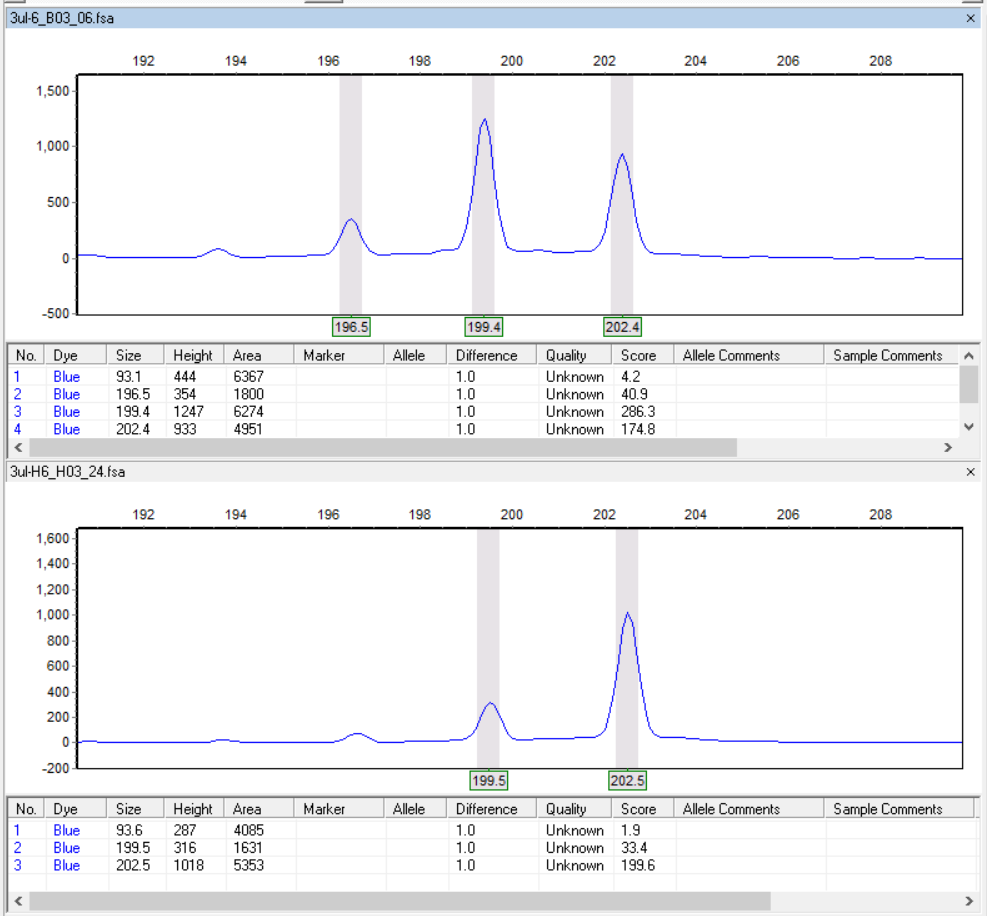

Supplement: Supplementary Materials — Supplementary Table S1: summary of laboratory results of our patient. Supplementary Figure S1: family pedigree and DNA sequencing chromatograms of the patient and his parents. Supplementary Figure S2: X-inactivation analysis of the patient's mother (MAGT1 carrier). [file 2390167.f1.zip › 2390167.f1/Supplementary Figure S2 (1).docx]
