# Supplementary material for: Novel MAGT1 Mutation Found in the First Chinese XMEN in Hong Kong
Source: Case Reports Immunol. 2022 Feb 14;2022:2390167. doi: 10.1155/2022/2390167 (PMC8860550; doi:10.1155/2022/2390167)
Supplement: Supplementary Materials — Supplementary Table S1: summary of laboratory results of our patient. Supplementary Figure S1: family pedigree and DNA sequencing chromatograms of the patient and his parents. Supplementary Figure S2: X-inactivation analysis of the patient's mother (MAGT1 carrier). [file 2390167.f1.zip › 2390167.f1/Supplementary Table S1 (1).docx]

**Supplementary Table S1. Summary of laboratory results of our patient**

| **Lymphocyte Subsets** | **Results (% of total lymphocytes and counts)** | **Reference ranges (% of total lymphocytes and counts)** |
| --- | --- | --- |
| B cells (CD19) % | 40.2% | 6.1-19.5% |
| B cells no. | 728/μL | 91-452/uL |
| T cells (CD3) % | 54.1% | 53.1-77.8% |
| T cells no. | 988/μL | 938-2311/μL |
| CD4 T cells % | 21.1% | 25.5-48.5% |
| CD4 T cells no. | 389/μL | 437-1226/μL |
| CD8 T cells % | 27.6% | 18.5-36.5% |
| CD8 T cells no. | 509/μL | 322-1104/μL |
| CD4:CD8 | 0.76 | 0.78-2.35 |
| NK cells (CD16/56) % | 5.8% | 8.4-36.6% |
| NK cells no. | 105/μL | 177-1059/μL |
|  |  |  |
| **B Cell Subsets** | **Results (% of total B cells)** | **Reference ranges (% of total B cells)** |
| Marginal zone B cells | 9.2 | 7.2-30.8 |
| Switched Memory B cells | 1.9 | 6.5-29.2 |
| Transitional B cells | 1.6 | 0.6-3.5 |
| Class-switched Plasmablast | 0.0 | 0.4-3.6 |
| CD21 Low B cells | 0.3 | 1.1-6.9 |
|  |  |  |
| **Immunoglobulins** | **Results (mg/dL)** | **Reference ranges (mg/dL)** |
| Ig G | 647 | 819-1725 |
| Ig A | 56 | 70-386 |
| Ig M | 114 | 55-307 |
|  |  |  |
| **Lymphocyte proliferation** | **Results (cpm/10^6 cells)** | **Reference ranges (cpm/10^6 cells)** |
| Unstimulated | 1667 | <3740 |
| PHA | 555440 | >236407 |
| Con A | 595200 | >70760 |
| PWM | 99447 | >35460 |
|  |  |  |
| **Liver function tests** | **Results (U/L)** | **Reference ranges (U/L)** |
| ALP | 80 | 42-110 |
| ALT | 134 | 8-58 |
| AST | 44 | 15-38 |
